# Neuropilin 1 expression correlates with the Radio-resistance of human non-small-cell lung cancer cells

**DOI:** 10.1111/jcmm.12623

**Published:** 2015-07-06

**Authors:** Juan Cong Dong, Hui Gao, Si Yao Zuo, Hai Qin Zhang, Gang Zhao, Shi Long Sun, Hai Ling Han, Lin Lin Jin, Li Hong Shao, Wei Wei, Shun Zi Jin

**Affiliations:** a Ministry of Health Key Laboratory of Radiobiology, Jilin UniversityChangchun, China; b China Institute for Radiation ProtectionTaiyuan, China

**Keywords:** ionizing radiation, neuropilin-1, non-small cell lung cancer, radio-resistance

## Abstract

The purpose of this study was to determine the correlation between over-expression of the neuropilin 1 (NRP1) gene and growth, survival, and radio-sensitivity of non-small cell lung carcinoma (NSCLC) cells. 3-[4,5-dimethylthylthiazol-2-yl]-2,5 diphenyltetrazolium broide (MTT) and colony assays were then performed to determine the effect of NRP1 inhibition on the *in vitro* growth of NSCLC cells. The Annexin V-Fluorescein Isothiocyanate (FITC) apoptosis detection assay was performed to analyse the effect of NRP1 enhancement on apoptosis of NSCLC cells. Transwell invasion and migration assays were employed to examine the metastatic ability of A549 cells post X-ray irradiation. In addition, Western blot assays were carried out to detect the protein level of VEGFR2, PI3K and NF-κB. Finally, to examine the effect of shNRP1 on proliferation and radio-sensitivity *in vivo*, a subcutaneous tumour formation assay in nude mice was performed. Microvessel density in tumour tissues was assessed by immunohistochemistry. The stable transfected cell line (shNRP1-A549) showed a significant reduction in colony-forming ability and proliferation not only *in vitro*, but also *in vivo*. Moreover, shRNA-mediated NRP1 inhibition also significantly enhanced the radio-sensitivity of NSCLC cells both *in vitro* and *in vivo*. The over-expression of NRP1 was correlated with growth, survival and radio-resistance of NSCLC cells *via* the VEGF-PI3K- NF-κB pathway, and NRP1 may be a molecular therapeutic target for gene therapy or radio-sensitization of NSCLC.

## Introduction

Lung cancer is the most common cause of cancer death, accounting for 26% of all female cancer deaths and 28% of all male cancer deaths 1. Non-small cell lung carcinoma (NSCLC) is the predominant type of lung cancer, accounting for approximately 80–85% in all lung cancers 2, and 70% of NSCLC patients have advanced disease at diagnosis, accompanied by distant metastasis, which reduces the chance of surgical resection and therapy is mainly radiotherapy alone or combined with chemotherapy 3. However, compared with SCLC, NSCLC shows more tolerance to radiotherapy 4. Thus, there is an urgent need to overcome the radio-resistance of NSCLC.

Angiogenesis promotes the growth and spread of tumours 5. A previous study reported that VEGF plays an important role in angiogenesis, however, clinical trials targeting the VEGF pathway are often ineffective, suggesting that other pathways are also important in tumour angiogenesis 6. Neuropilin 1 (NRP1), a non-tyrosine kinase transmembrane protein, functions as a co-receptor for VEGF, suggesting that NRP1 may have an important role in tumour angiogenesis 7. The report showed that NRP1 played a key role in newly sprouting vessels 5. Neuropilin 1 transgenic mice have excessive capillaries and blood vessels 8, whereas NRP1 knockout mice display severe defects in vascular development 9, suggesting that NRP1 plays a role in vasculogenesis.

Neuropilin 1 is expressed by a wide variety of human tumour cell lines and diverse human neoplasms, and is implicated in mediating the effects of VEGF on the proliferation, survival and migration of cancer cells 10. Neuropilin 1 was extensively expressed in tumour vasculature, where NRP1 over-expression is associated with tumour progression and poor clinical outcome 11. Shokoufeh reported that ultraviolet radiation B (UVB) irradiation of mouse skin induced NRP1 up-regulation 12. Our previous study indicated that X-rays can induce NRP1 to upregulate T regulatory cells 13, which play an important role in tumour immunosuppression. In addition, mice lacking NRP1 in the epidermis were more sensitive to UVB irradiation and displayed increased apoptosis following irradiation 14, suggesting that increased NRP1 in the epidermis is necessary for their survival. These reports show that NRP1 may increase the radio-resistance of tumour cells. Kawakami *et al*. demonstrated in 2002 that the expression level of NRP1 gene in neoplastic tissue was higher than extraneoplastic lung tissue, and 55 of 68 NSCLC specimens showed NRP1+ gene expression (80.9%) 15. Hong *et al*. reported that Patients with high NRP1 expression had shorter disease-free and overall survival compared with low NRP1-expression patients 16. To date, however, there has been no association between NRP1 over-expression and radio-resistance of NSCLC cells, or whether NRP1 could be a molecular target for radiosensitization of NSCLC.

To clarify the potential function of NRP1 in the radio-sensitivity of NSCLC, we attempted to use small interfering RNA (siRNA) technology to inhibit expression of the NRP1 gene in NSCLC cells (A549) and analysed the effect of NRP1 inhibition on growth, survival and radio-sensitivity of NSCLC cells both *in vitro* and *in vivo*. Here, we demonstrate that NRP1 receptors are highly expressed in lung cancer. Furthermore, knockdown of NRP1 expression enhanced radio-sensitivity. These results suggest that NRP1 could function as a biomarker of radio-resistance in NSCLC.

## Materials and methods

### Cell lines and reagents

The cell lines (HepG2, MCF-7, U87, PC-3, A549, H460, H358, H1299 and SK-MES-1 were obtained from the Type Culture Collection of the Chinese Academy of Sciences. Cell lines were cultured in DMEM (Gibco, Beijing, China, USA) or RPMI medium 1640 (Gibco) supplemented with 10% (vol/vol) fatal bovine serun (FBS) (HyClone, Beijing, China, USA) and 1% penicillin-streptomycin. Human anti-VEGFR2 antibody and anti-NRP1 antibody were from Abcam (Cambridge, MA, USA). Anti-PI3K antibody and anti-NF-κB antibody were from Cell Signaling Technology (Beverly, MA, USA). Anti-GAPDH antibody was from Santa Cruz Biotechnology (Santa Cruz, CA, USA). The cell lysis and secondary antibodies (goat anti-rabbit) were bought from Beyotime (Shanghai, China).

### Animals

The mice were maintained in a specific pathogen-free facility and were housed in micro-isolator cages containing sterilized feed, autoclaved bedding and water. All experimental manipulations were undertaken in accordance with the Institutional Guidelines for the Care and Use of Laboratory Animals. All mice had a severe combined immunodeficiency(SCID) background.

### Construction and transfection of lentiviral vectors with specific shRNA for NRP1

Construction of the pSIREN–RetroQ–NRP1 short hairpin (sh) RNA plasmid was performed as previously described 17, and contained the 19 bp target sequence for NRP1 (5′-GATCGACGTTAGCTCCAAC-3′). Recombinant retroviruses were packaged using GP2-293 cells according to the BD Retro-XTM Universal Packaging System protocol (Clontech, Toyobo Osaka, Japan, USA). Forty-eight hours later, the supernatant was collected and tittered by serial dilutions. The supernatant containing the optimal concentration of viral particles was used for the knockdown experiments.

### Irradiation protocol

Cells were sham-irradiated or exposed to ionizing radiation (IR) of 10 Gy which was delivered at the dose rate of 0.341 Gy/min. by an X-ray generator (Dandong City Yasuyoshi Equipment Co, Ltd, Jilin, China). Adult (8-week-old) male SCID mice were exposed to a single 20 Gy dose of X rays, the dose rate was 1.55 Gy/min. Age-matched, unirradiated mice served as normal controls.

### Quantitative reverse transcriptase-polymerase chain reaction

qRT-PCR was performed to determine the expression of NRP1 mRNA. Briefly, total RNA was extracted from frozen tissue and cells by homogenization with a power homogenizer in Trizol Reagent (Invitrogen, Carlsbad, CA, USA) according to the manufacturer’s protocol and reverse transcribed to generate cDNA (PrimeScript RT-PCR kit; Takara Bio, Toyobo Osaka, Japan). GAPDH was used as an internal control. The levels of mRNA encoding were quantified by real-time PCR with the Applied Biosystems (California, USA) Mx3000P, real-time PCR System using SYBR Premix Ex Taq (Applied Takara Bio, Toyobo Osaka, Japan). The sequences of the primers were as follows: All qRT-PCRs were performed in duplicate. Relative quantification of NRP1 mRNA expression was calculated using the 2^−ΔΔCT^ method.

### MTT analysis

Cells were individually seeded into 96-well plates at a concentration of 5 × 10^3^ per well filled with medium which was removed and replaced with fresh medium. The cells received X-rays at different doses, and were then cultured at 37°C in a CO_2_ incubator for 48 hrs before the assays. The assays were initiated by adding 20 μl MTT (5 mg/ml) to each well and incubating the cells for an additional 4 hrs. Finally, the medium was removed and 150 μl dimethylsulphoxide was added to each well. Plates were read at a wavelength of 570 nm.

### Colony formation assays

According to the dose of irradiation, different numbers of cells were seeded in six-well plates. Cells received X-rays at the dose of 0, 2, 4, 6, 8 or 10 Gy and the cells were then cultured at 37°C in a CO_2_ incubator for 14 days to allow colonies to grow. The cells were then rinsed with PBS, fixed in methanol, and stained for 30 min. with Giemsa. Finally, the formation rate of the cell clone was calculated.

### Annexin V-FITC apoptosis detection assay

After treatment, the cells were prepared and stained with the Annexin V-FITC Apoptosis Detection Kit I (BD Biosciences, San Jose, CA, USA) according to the manufacturer’s instructions. Subsequently, 10,000 cells per treatment were counted to assess the positive staining cells.

### Western blot analysis and immune-precipitation

After treatment, cell lysates were prepared and then separated by SDS-PAGE gels. Following the transfer, PVDF membranes were incubated with the designated primary antibodies overnight in a cold room at 4°C. Subsequently, bound primary antibodies were reacted with corresponding secondary antibodies for 2 hrs at room temperature and detected by chemiluminescence. For immune-precipitation, cell lysates were first precipitated with an antibody. The immune-precipitates were then separated on a SDS-PAGE gel for immune-blotting.

### Transwell assay

Invasion assays were performed in triplicate using 24-well microchemotaxis chambers with 8-μm pore membranes (Millipore, Bedford, MA, USA) pre-coated with 10 μg/ml Matrigel (BD Biosciences). Cells were divided into four groups: control group, IR group, shNRP1 group and shNRP1+IR group. The bottom chamber was filled with 10 μg Fibronectin (BD Biosciences)/DMEM, and the cells were suspended in DMEM containing 10% NuSerum, and 5 × 10^4^ cells were placed in the upper well of each chamber. A549 cells treated with 10 Gy X-rays were incubated at 37°C for 12 hrs. The suspended media in the lower chamber were removed. The cells which had invaded the lower side of the filter were fixed in 4% paraformaldehyde and stained with Giemsa solution. The number of cells that passed through the pores into the lower chamber was counted under a phase-contrast microscope. Each sample was assayed in triplicate.

The migration capability of A549 cells was determined from the Transwell migration chamber as described above. However, the polycarbonate membranes (containing 8-Am pores) of Transwell inserts were not coated with Matrigel. The cells were suspended in DMEM containing 10% NuSerum, and 5 × 10^3^ cells were placed into the upper well of each chamber.

### Tumour xenografts

Approximately 1 × 10^6^ untransfected or stably transfected A549 cells were inoculated s.c. with 0.2 ml PBS buffer into the flank region of 8-week-old male SCID/nude mice. When the tumours grew to 50 mm^3^ in diameter, the mice were then exposed to X-rays, and received 20 Gy of irradiation. The mice were killed 15 days after irradiation, and the tumour mass and volume were recorded. Volume was calculated as (length/2) × (width^2^). The NRP1 protein expression in tumours was then detected by western blot analysis as described above.

### Immunohistochemistry

The expression of NRP1 in mouse tumour tissues was examined by immunohistochemistry. The samples were fixed with formalin, embedded in paraffin and cut into 4-μm-thick sections. The sections were then dewaxed in xylene and rehydrated with graded concentrations of ethanol. After blocking with 0.3% hydrogen peroxide, the antigens were retrieved in a microwave in 10 mM citrate buffer (pH 6.0) for 30 min. and cooled to room temperature. After washing with PBS, the sections were incubated overnight at 4°C with mouse monoclonal antibody against human NRP1 at a dilution of 1:300 (Abcam). Subsequently, horseradish peroxidase conjugated secondary antibody was used. The sections were developed with diaminobenzidine tetrahydrochloride and counterstained with hematoxylin. Negative controls were included in which the primary antibody was replaced by PBS. The degree of immunostaining was evaluated by two independent observers who were blind to the clinical data of the patients.

### Statistical analyses

Data were expressed as mean ± SD. All statistical analyses were performed using the SPSS 16.0 statistical software (SPSS Company, Chicago, IL, USA). Differences between the control and experimental groups were analysed using one-way anova between groups, and *P* < 0.05 was considered statistically significant.

## Results

### NRP1 expression correlates with the radio-resistance of human NSCLC cells

**Figure 1:**
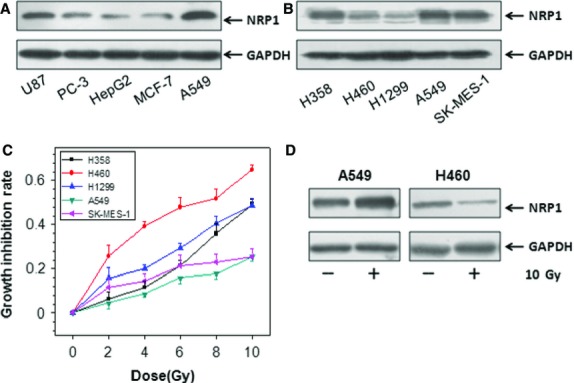
The expression of NRP1 and the radio-sensitivity of NSCLC cells. (A) The protein level of NRP1 was determined by immunoblotting in U87, PC-3, HepG2, MCF-7, A549 cells. (B) The protein level of NRP1 was determined by immunoblotting in 5 NSCLC cell lines (H358, H460, H1299, A549, SK-MES-1). (C) The cells were exposed to different doses of X-rays, and growth inhibition was examined by MTT assay 24 hrs after irradiation. (D) The change in NRP1 expression was measured after 10 Gy X-rays.

The expression of NRP1 protein was detected and analysed in different tumour cell lines (U87, PC-3, HepG2, MCF-7, A549), and the results showed that the protein expression level of NRP1 was different in various tumour cell lines and was significantly higher in A549 cells, compared to other tumour cells (Fig.1A). Next, we examined the expression level of NRP1 in five NSCLC cell lines (H358, H460, H1299, A549, SK-MES-1), and compared their inhibitory effect after 10 Gy X-rays. We found that NRP1 levels were highest in A549 cells (Fig.1B). The inhibitory effect of 10 Gy X-rays on the proliferation of A549 tumour cells was significantly lower than that of other tumour cells, and the inhibitory effect in H460 tumour cells was highest. We further investigated the possible relationship between NRP1 and NSCLC by evaluating the radio-sensitivity of A549 and H460 cells. The results showed that colony formation in A549 cells was higher than that in H460 cells after 10 Gy X-rays (Fig. S1), and the apoptosis of H460 cells was significantly higher than that of A549 cells. It is noteworthy that NRP1 expression was up-regulated in A549 cells after radiation and decreased in H460 cells (Fig.1D). Our results demonstrated that high expression of NRP1 was frequently observed in A549 cells. In contrast, H460 cells showed lower NRP1 expression. These findings suggest that the up-regulated expression of NRP1 may provide a selective advantage in the radio-resistance of human NSCLC.

### Relative expression of NRP1 in A549 cells

**Figure 2:**
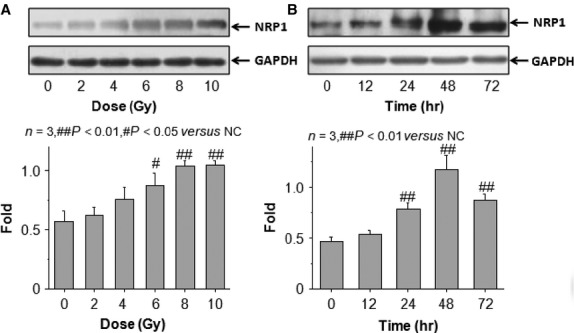
Relative expression of NRP1 in A549 cells following X-ray irradiation. (A) Dose-effect change. (B) Time-effect changes.

Next we monitored the kinetics of NRP1 expression in A549 cells after different doses of X-rays (2, 4, 6, 8 and 10 Gy) and at different time-points (12, 24, 48 and 72 hrs) after exposure to 10 Gy X-rays. The data in Figure2A show that, compared with the control group, the expression of NRP1 showed a dose-dependent increase, and reached a peak at 10 Gy X-rays. The NRP1 protein was up-regulated 24 hrs post-irradiation and remained at a high level up to 72 hrs post-irradiation. The relative NRP1 expression 48 hrs post-irradiation with 10 Gy X-rays was significantly higher than that at other time-points (Fig.2B). Thus, we selected 10 Gy X-rays and determined the effect of this dose at 48 hrs after irradiation.

### Endogenous NRP1 expression knockdown enhances the radio-sensitivity of A549 cells

**Figure 3:**
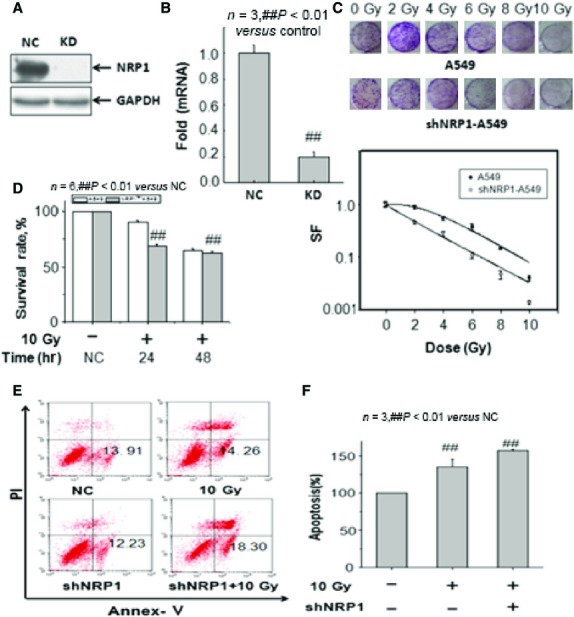
Analysis of the effect of siRNA-mediated NRP1 inhibition on the *in vitro* radio-sensitivity of NSCLC cells. (A) Transfection efficiencies of the NRP1 shRNA lentivirus (KD group) and empty lentivirus (NC group). The NRP1 gene was knocked down by NRP1 shRNA lentivirus. Western blot assay demonstrated that, normalized by GAPDH, NRP1 protein expression was degraded in the KD group *versus* the NC group. (B) Real-time PCR showed a significant decrease in NRP1 mRNA (by 80%) in the KD group *versus* the NC group. (C) Clonogenic survival of untransfected or stably transfected A549 cells. Compared with control cells, shNRP1-A549 cells showed significantly lower clonogenic survival. (D) The MTT assay showed that RNAi-specific to NRP1 led to a marked reduction in the survival of A549 cells after irradiation. (E and F) Apoptosis was determined by the Annexin V assay. The apoptotic rates of shNRP1-A549 cells treated with irradiation (10 Gy) were significantly increased compared with control cells treated with irradiation (10 Gy).

To determine the importance of NRP1 in radio-resistance of A549 cells, shNRP1 was introduced into the lung cancer cells. shRNA efficiently knocked down NRP1. In the western blot assay, NRP1 protein expression was markedly inhibited in the NRP1 shRNA lentivirus (KD) group compared to the empty lentivirus (NC) group, and the real-time PCR results demonstrated that NRP1 shRNA had a significant suppressive effect. The NRP1 mRNA level in the KD group decreased by 80% compared to the NC group (Fig.3A and B). MTT and colony assays showed that RNAi-specific to NRP1 led to a marked reduction in the proliferation and colony-forming ability of A549 cells after irradiation (Fig.3C and D).

The Annexin V assay was performed to determine apoptosis of the untransfected or stably transfected NSCLC cells treated with irradiation (Fig.3E and F). The apoptotic rates of shNRP1 A549 cells treated with 10 Gy irradiation were significantly increased compared with A549 cells treated with 10 Gy irradiation. Thus, RNAi-mediated NRP1 inhibition may enhance the radio-sensitivity of NSCLC cells by increasing radiation-induced apoptosis. These data show that inhibition of NRP1 expression by shNRP1can significantly enhance the *in vitro* radio-sensitivity of NSCLC cells.

### NRP1 blockade leads to NSCLC regression

**Figure 4:**
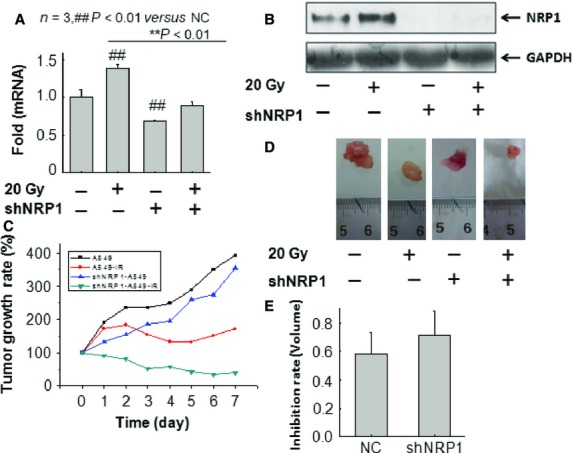
In vivo evaluation of radio-sensitivity in untransfected or stably transfected A549 xenografts. (A and B) Western blot and real-time PCR analysis of NRP1 expression in tumour tissues from each group of mice. The levels of NRP1 protein were significantly downregulated in tumour tissues from sh-A549 cells. (C) The growth of tumours was assessed by measuring tumour volume. The growth of tumours formed from shNRP1-A549 cells treated with irradiation developed slower than tumours from control cells treated with irradiation. (D and E) At d22, the mice were killed and the tumour volume was calculated. The volume of tumours from shNRP1-A549 cells treated with irradiation was significantly reduced by approximately 13% compared with that of tumours formed from A549 cells.

A subcutaneous (s.c.) tumour formation assay in nude mice demonstrated that the tumours formed from shNRP1-A549 cells developed slower than the tumours developed from untransfected A549 cells. qRT-PCR and Western blot assays indicated that the expression levels of NRP1 mRNA and protein were significantly lower in tumours from shNRP1-A549 cells than in tumours from A549 cells (Fig.4A and B). Thus, RNAi-mediated inhibition of NRP1 induced *in vivo* proliferation inhibition of NSCLC cells.

Experimental radio-gene therapy in a nude mouse s.c. tumour model was performed. Briefly, the stably transfected NSCLC cells were subcutaneously injected into the right flank of nude mice, and the mice were treated with either no radiation or radiation alone. When the mice were irradiated with 20 Gy X-rays, the growth of tumours formed from shNRP1 to A549 cells treated with irradiation was significantly delayed compared with that of tumours formed from A549 cells treated with irradiation (Fig.4C). The volume of tumours from shNRP1 to A549 cells treated with irradiation on d22 was significantly reduced by approximately 13% compared with that of tumours formed from A549 cells treated with irradiation (Fig.4D and E). These results show that NRP1 inhibition combined with radiotherapy can induce a stronger *in vivo* anti-tumour effect than radiotherapy alone.

### Blockade of NRP1 has inhibited cell invasion and Angiogenesis after irradiation

**Figure 5:**
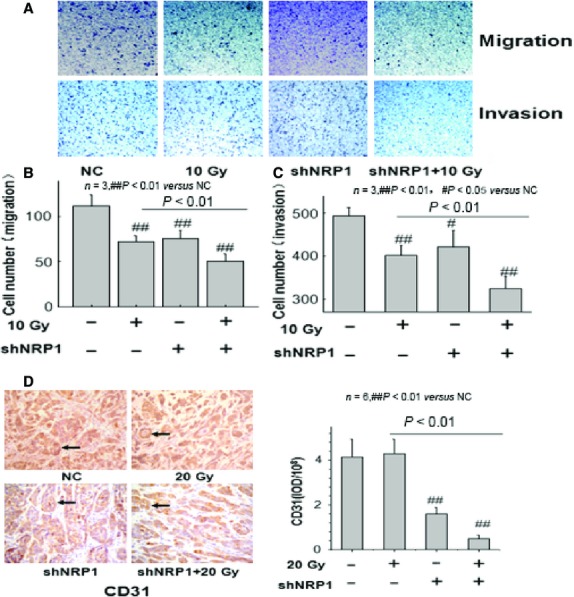
(A–C) The effect of X-ray irradiation on the migration and invasion of A549 cells *in vitro*. A549 cells treated with 10 Gy X-rays, shNRP1 and shNRP1 + 10 Gy X-rays were allowed to migrate on the Transwell inserts for 12 hrs, and A549 cells without X-ray irradiation acted as the control group. The cells which invaded and migrated to the lower side of the filter were fixed in 4% paraformaldehyde and stained with Giemsa solution. The number of cells that passed through the pores into the lower chamber was counted under a phase-contrast microscope. (D) Microvessel density (MVD) of xenograft tumours in the knockdown (KD), negative control (NC) (magnification, ×200). NRP1 silencing led to a significant decrease in MVD in the KD group.

The results in Figure5A–C show that compared with unirradiated A549 cells, the invasiveness and migration of A549 cells irradiated by 10 Gy X-rays decreased significantly. Interestingly, shNRP1 significantly decreased the number of cells which invaded and migrated after irradiation. Microvessel density (MVD) was determined by anti-CD31 antibody staining. As the results of the immunohistochemical analysis showed, shNRP1 had a suppressive effect on the neovascularization and angiogenesis of tumours. The MVD in the KD group was lower than that in the NC group, while shNRP1 significantly inhibited the MVD after irradiation. In conclusion, NRP1 is a potential target for anti-angiogenic therapy in NSCLC.

### NRP1 directly activates pro-survival pathways in NSCLC after irradiation

**Figure 6:**
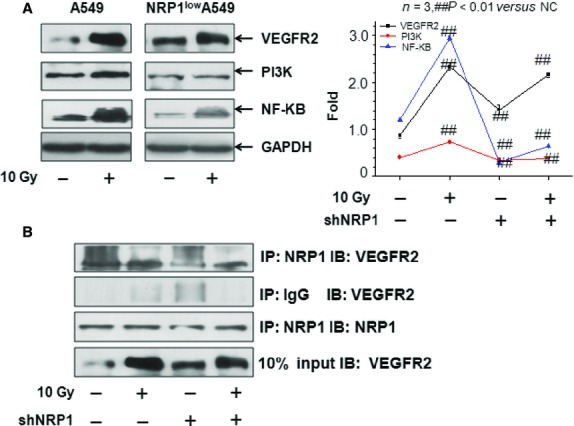
(A) The expression levels of NRP1, VEGFR2, PI3K, and NF-κB in A549 cells and shNRP1-A549 cells. The expression levels of NRP1, VEGFR2, PI3K, and NF-κB in shNRP1-A549 cells 48 hrs after 10 Gy X-rays were significantly lower than those in A549 cells. (B) Immunoprecipitation (IP)/Western blotting (WB) of VEGFR2 and NRP1 in A549 and shNRP1-A549 cells pre-treated or not with 10 Gy X-rays.

Ionizing radiation-induced cellular survival signalling pathways result in the development of cancer and insensitivity of tumour cells to radiation therapy. Neuropilin 1 was shown to serve as a co-receptor for VEGF, and NRP1 forms complexes with VEGFR2 to enhance the binding of VEGF to VEGFR2 and promotes VEGF165-mediated tumour angiogenesis, cell migration and tumourigenicity 18. Therefore, we investigated the protein VEGFR2 level in A549 cells and shNRP1-A549 cells, and the expression of PI3K and NF-κB. The results showed that lentivirus-mediated knockdown of NRP1 resulted in a significant decrease in VEGFR2, PI3K and NF-κB expression in shNRP1-A549 cells and these decreases were lower than those in A549 cells. These results indicated that combining shNRP1 and irradiation may increase the radio-sensitivity of A549 cells *via* upregulated VEGFR2, PI3K and NF-κB in A549 cells (Fig.6A).

To further examine the potential role of NRP1 in VEGF-VEGFR2 signalling in A549 cells following irradiation, we compared the co-immunoprecipitation of VEGFR2 and NRP1 in extracts from A549 cells that were untreated, treated with 10 Gy X-rays, treated with shNRP1 or with shNRP1+10 Gy X-rays. As shown in Figure6B, treatment with irradiation not only up-regulated NRP1 expression, but increased the interaction between VEGFR2 and NRP1.

## Discussion

Radiotherapy is a common strategy in treating NSCLC; however, the survival ratio cannot be enhanced efficiently by radiotherapy due to radiation resistance. Thus, a novel strategy is needed to overcome radiation resistance in human NSCLC.

Neuropilin 1 was first described as a semaphoring receptor important for the guidance of developing neurons 19. It is a transmembrane glycoprotein that acts as a co-receptor for a number of extracellular ligands including class III/IV semaphorins, certain isoforms of VEGF and transforming growth factor beta 1. There is mounting evidence to indicate that NRPs have alternative roles in tumour biology, such as modulating the balance between cell proliferation and survival. Neuropilin 1 has been observed to be expressed in patient specimens from lung, breast, prostate, pancreatic and colon carcinoma, but not in corresponding normal epithelial tissues 10. The over-expression of NRP1 enhances tumour growth, correlates with invasive growth and is associated with poor prognosis in gastrointestinal tract, prostate, lung and ovarian tumours as well as gliomas, osteosarcomas and melanomas 15,20,21. Preclinical data suggest that blockade of NRP1 suppresses tumour growth by inhibiting angiogenesis or by impairing survival and proliferation in a variety of cancer cell types 16. As for its important roles in tumourigenesis and tumour progression, NRP1 has the potential to be a good therapeutic molecular target in cancer therapy.

Although the expression level of NRP1 in NSCLC was higher than the level of NRP1 in corresponding extra-neoplastic lung tissue, it was significantly correlated with a poor prognosis in patients with NSCLC due to neovascularization 15, and there have been no reports on a correlation between NRP1 activation and radio-resistance in NSCLC cells. To determine whether NRP1 could be a molecular therapeutic target or radiosensitizer in the treatment of NSCLC, we employed RNA interference technology to inhibit expression of the NRP1 gene and analyse the effect of NRP1 inhibition on growth, survival and radio-sensitivity of NSCLC cells. As NRP1 is highly expressed in A549 cells, we selected A549 cells in the present study. Neuropilin 1 inhibition significantly inhibited the growth of NSCLC cells not only *in vitro*, but also *in vivo*. The growth inhibition of NSCLC cells due to NRP1 inhibition may be associated with the enhancement of apoptosis. This is in agreement with the proposal that a small molecule ligand that inhibits VEGF-A binding to NRP1 reduces the viability of A549 lung carcinoma cells *in vitro* 22. Neuropilin 1 knockdown using siRNA inhibited breast carcinoma cell migration 23, and a peptide targeted to the VEGF binding site of NRP1 induced breast tumour cell apoptosis 24. Moreover, we first reported that RNAi-mediated NRP1 inhibition could enhance radio-sensitivity of NSCLC cells both *in vitro* and *in vivo*. This knowledge has facilitated the clinical study of numerous biological modifiers that may increase the potency of treatment and reduce undesirable side effects through the enhancement of radio-sensitivity of tumour cells, thereby helping to optimize the radiation dose aimed at strategic improvement of the therapeutic outcome. Therefore, siRNA or a tumour selective cytotoxic agent might be used to overcome the development of radio-resistance in NSCLC.

Considerable experimental evidence has shown that NRP1 plays an essential role in tumour growth and metastasis by regulating angiogenesis. Several studies have demonstrated that a vascular supply is required to support the growth of tumours beyond 1–2 mm^3^ (reviewed in Ref. 25). Tumour growth depends on the formation and expansion of a functional vasculature to distribute oxygen and other blood constituents. Neuropilin 1 has been reported to be associated with the development of the cardiovascular system 26. Blockade of NRP1 activity represents a novel anti-angiogenic strategy as it not only inhibits angiogenesis, but also vascular remodelling, potentially by altering pericyte function 27. It is unclear how NRP1 expression is involved in the progression of NSCLC through vascularization. Recent studies provide direct evidence that a peptide which inhibits VEGF binding to NRP1 has been reported to inhibit angiogenesis and the growth of tumour xenografts 28. In the current study, we analysed expression of the NRP1 gene and angiogenesis in NSCLC. The results indicated that animals with NRP1 deficiency exhibited lower MVD in NSCLC xenografts, and combining shNRP1 with irradiation treatment resulted in the synergistic inhibition of intratumoural vessel formation to promote tumour growth inhibition. Neuropilin 1 up-regulation in gastrointestinal carcinomas appears to correlate with invasive behaviour and metastatic potential 29. Neuropilin 1 expression leads to enhanced migration and survival of ECs *in vitro* 30. Neuropilin 1 also appears to be preferentially expressed in metastatic cells, and is found, for example, in the metastatic breast cancer MDA-MB-231 and melanoma MDA-MB-435 cell lines, but not in the non-metastatic cell line MDA-MB-453 or in some non-metastatic tumours 31,32. Antibodies which specifically block VEGF binding to NRP1 inhibited the migratory response to VEGF 33. Interestingly, in this study we found that blocking NRP1 by shNRP1 prevented cell invasive and migration. Moreover, shNRP1 and irradiation treatment resulted in a synergistic inhibitory effect.

**Figure 7:**
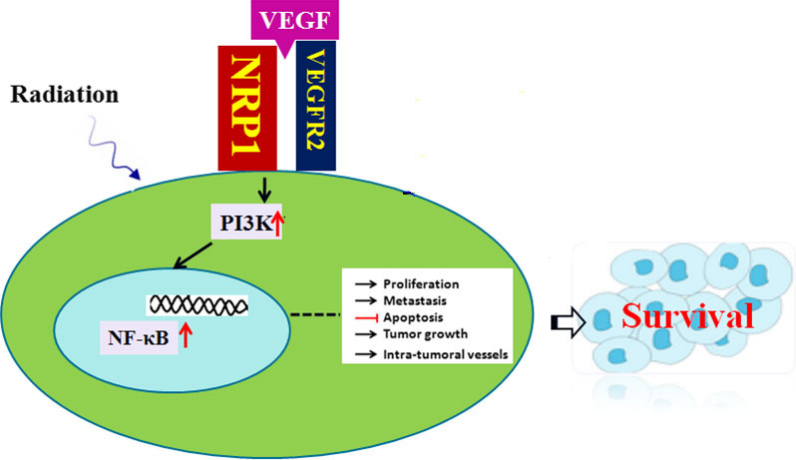
A model of NRP1 up-regulation of radio-resistance in human NSCLC *via* the VEGFR2-PI3K-NF-κB pathway.

In light of the fact that the NRP1 receptor does not have intrinsic kinase activity, the function of NRP1 in tumour cells requires more detailed analysis of the signal transduction pathways. The majority of studies support that NRP1 functions as a co-receptor of VEGF and enhances vascular VEGFR2 activity in the presence of VEGF 21. siRNA-mediated NRP1 knockdown resulted in 50% inhibition of VEGFR2 phosphorylation at Tyr^1175^ 34,35, a residue required for VEGFR2-mediated activation of phospholipase C-γ/extracellular signal-regulated kinase (ERK), cell proliferation and normal embryonic development. To determine the potential role of NRP1 in VEGF-VEGFR2 signalling in A549 cells following irradiation, we detected a change in the interaction between VEGFR2 and NRP1 in a ligand-dependent manner, and the results showed that treatment with irradiation not only up-regulated NRP1 expression, but increased the interaction between VEGFR2 and NRP1. However, the VEGFR2-NRP1 complex was significantly decreased after irradiation and shNRP1. Beck reported that the VEGF-VEGFR2-NRP1 signalling axis was implicated in generating a perivascular niche for proliferation of cancer stem cells in squamous cell skin tumours 36. Considerable experimental evidence has suggested that cancer stem cells were more radio-resistant. In addition, Shi *et al*. showed that knocking down VEGFR2 by MiR-200c could increases the radiosensitivity of non-small-cell lung cancer cell line A549 37. Thus, these results suggested that NRP1 could increase radio-resistance *via* VEGF-VEGFR2-NRP1 signalling. A previous report showed that blocking NRP1 function with siRNA or anti-NRP1 antibody treatment decreased mammosphere formation by breast cancer cells *in vitro via* NF-κB signalling 38. In the present study, we also detected a change in NF-κB following irradiation or shNRP1, and the results indicated that lentivirus-mediated knockdown of NRP1 resulted in a significant decrease in NF-κB expression in shNRP1-A549 cells which was lower than that in A549 cells. In addition, PI3K expression in shNRP1-A549 cells was also lower than that in A549 cells after irradiation. Taken together, these results suggest that NRP1 can increase radio-resistance *via* the VEGFR2-PI3K-NF-κB pathway (Fig.7). Mathieu *et al*. found that the expression of cyclooxygenase-2 (COX-2), prostaglandin E synthetase (PGES), lung-related resistance protein (LRP) and glutathione-S-transferase (GSTs) could rendered the chemo-resistant of A549 NSCLC orthotopic xenografts, and these molecular expression were correlated with NSCLC prognoses 39, thus we speculated that these molecules may also be associated with the radiation resistance of NSCLC, but it was needed experiments to clarify.

In conclusion, shNRP1 combined with radiation sensitized A549 cells *in vitro*. The radiosensitization induced by shNRP1 may be mediated by the VEGFR2-PI3K-NF-κB - pathway. Our results provide novel insights into this area of NSCLC research. This study demonstrated that the level of expression of NRP1 was highly increased in A549 cells, and over-expression of NRP1 may play an important role in the radio-resistance of human NSCLC. A better understanding of NRP1 signalling may enable the design of therapies that could improve the radio-sensitivity of NSCLC, and NRP1 expression should be helpful in predicting the radio-resistance of patients with NSCLC. Our studies provide data on the molecular mechanisms underlying the NRP1-induced increase in radio-resistance in human NSCLC. However, the exact molecular mechanism requires further study.

The results of the current study show that the A549 NSCLC orthotopic model in nude mice is resistant to irradiation. The high levels of radioresistance observed in the A549 NSCLC orthotopic xenografts can be explained at least in part by the high levels of expression of NRP1. Thus, the level of NRP1 expression will now be used to screen the patients with NSCLC, and the patients who express the low level of NRP1 could select the radiotherapy.

## Supporting Information

**Figure S1** Analysis of the radio-sensitivity of A549 cells and H460 cells (A) Clonogenic survival of A549 cells and H460 cells. (B) The apoptotic rates of A549 cells and H460 cells. Apoptosis was detected by the Annexin V assay.
