# Supplementary figures and images for: Neuropilin 1 expression correlates with the Radio-resistance of human non-small-cell lung cancer cells
Source: J Cell Mol Med. 2015 Jul 6;19(9):2286–95. doi: 10.1111/jcmm.12623 (PMC4568932; doi:10.1111/jcmm.12623)

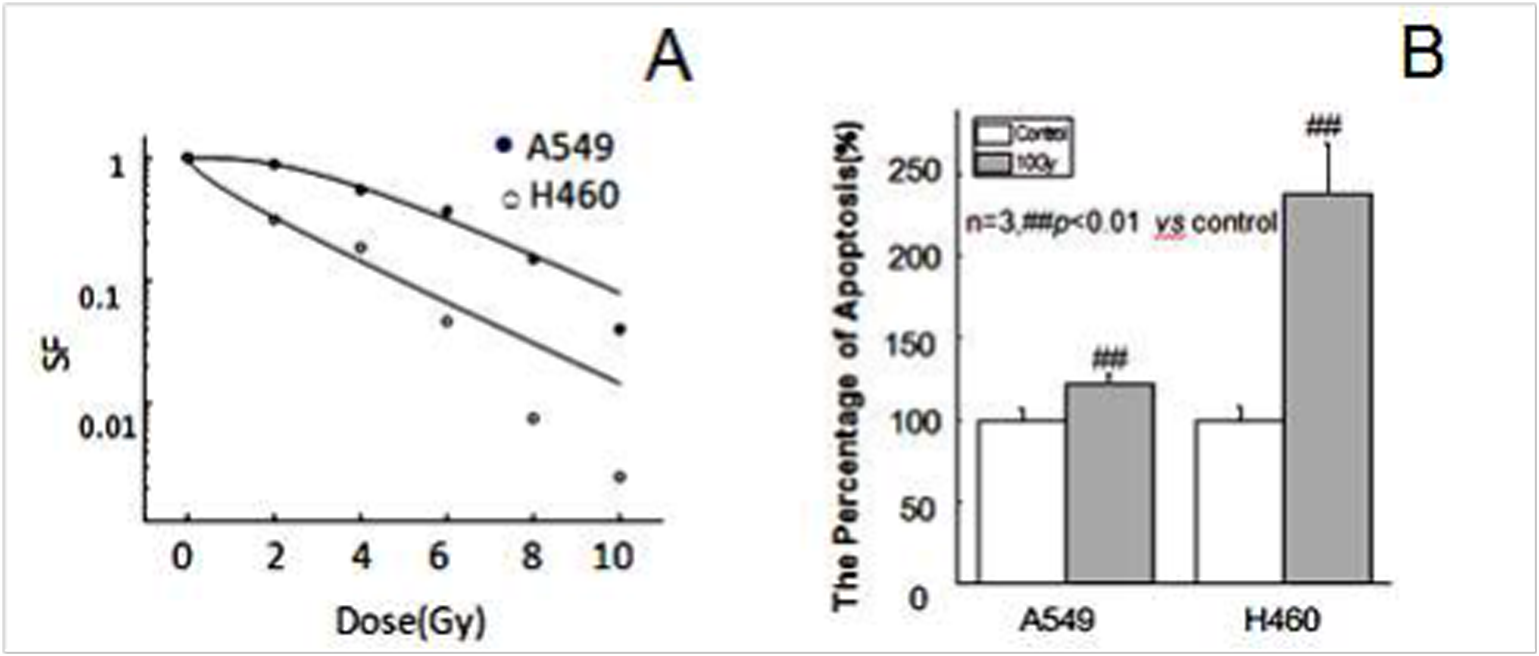

Supplement: Supplementary file 1 [file jcmm0019-2286-sd1.tif]
